# Video-assisted pulmonary lobectomy combined with transmanubrial approach for anterior Pancoast tumor resection: case report

**DOI:** 10.1186/s13019-016-0446-7

**Published:** 2016-04-14

**Authors:** Lorenzo Rosso, Alessandro Palleschi, Paolo Mendogni, Mario Nosotti

**Affiliations:** Thoracic Surgery and Lung Transplant Unit, Fondazione IRCCS (Scientific Institute for Research Hospitalization and Health Care) “Ca’ Granda” General Hospital University of Milan, Via Francesco Sforza, 35, Milan, 20122 Italy

**Keywords:** Anterior Pancoast, VATS lobectomy, Lung cancer, Chest wall, Transmanubrial approach

## Abstract

**Background:**

The mini-ivasive approach to superior sulcus tumors is an uncommon procedure that is still far from standardization. We describe a hybrid surgical technique to approach “en block” chest resection and pulmonary lobectomy for anterior superior sulcus tumors.

**Case Presentation:**

A patient affected by right anterior Pancoast tumor surgically staged as cT4N0M0 (suspected anonymous vein invasion) underwent chemo-radiation induction therapy with satisfactory tumor reduction. The surgical operation comprised an initial VATS approach to the hilar structures followed by a limited C-shaped anterior contra-incision; finally, the right upper lobe “en block” with the anterior part of the first and second rib was removed. The whole procedure was conducted with the patient in the supine position; no rib retractors were used. The definitive stage was ypT0N0M0. The patient had an uneventful hospital stay and at the 9 months follow-up she was free from disease and post-thoracotomy syndrome.

**Conclusions:**

In our opinion such hybrid VATS procedure has several advantages: starting with thoracoscopy it is possibleto exclude previously undetected pleural dissemination and to precisely define the tumor location as well as limits of the thoracic wall resection; time could be spared maintaining the patients in the supine position for both surgical times; postoperative pain and post-thoracotomy syndrome could be minimized avoiding the use of any rib retractor.

## Background

Thoracoscopic “en bloc” chest wall resection is a procedure not fairly usual for Video-assisted thoracoscopic surgery (VATS) lobectomy; this report describes technical details of an emerging new surgical procedure consisting in a hybrid technique for treatment of anterior Pancoast tumor.

Despite the increasing acceptance of VATS lobectomy even for advanced surgical procedures, anterior Pancoast tumors have been rarely approached with VATS combined with transmanubrial approach [1]. The first case was described by Truin in 2010, afterwards a restricted number of cases approached with hybrid procedures were reported [2–5]. In these series (6 patients), the induction therapies as well as free intervals were heterogeneous, nevertheless the follow-up was encouraging (Table 1). From the technical point of view, only Yokoyama managed the apex via the transmanubrial approach as the second surgical step as we did. The vast majority of patients underwent first rib resection; moreover, three patients had an additional bone resection. T1 root or vascular invasion management was necessary in three cases. The mean hospital stay was 13.2 days while morbidity was present in 40% of the cases.

**Table 1:** Reported clinical experiences

Author	G.	Age	Pathol.	Clinical stage	Induction therapy	Free Interval (weeks)	Technique	Patient position	Extended resection	Pathol. stage	LOS (days)	Complications	DFI (Months)	Alive
Truin	F	60	NSCLC	cT3N0M0	Cisplatin + etoposide	6	Apex first	Supine + Lateral	1^st^ rib + T1 root + Subcl. vein	ypT0N0M0	7	Right arm mild edema	NA	NA
Nakajima	M	59	NSCLC	cT3N0M0	Cisplatin + Mitomycin C + Vindesine +45 Gy	6	Apex first	Supine	1^st^ rib + T1 root + Scalene m.	ypT0N0M0	NA	Left lung atelectasis Phrenic nerve paralysis	NA	NA
Shikuma	M	59	NSCLC	cT3N0M0	Cisplatin + docetaxel + 60 Gy	NA	Apex first	Supine + Lateral	None	ypT3N0M0	NA	None	24	yes
Yokoyama	M	79	Pleom. Carc.	cT3N0M0	None	NA	VATS first	Lateral + Supine	1^st^ + 2^nd^ ribs	pT3N0M0 R1	18	None	58	yes
	M	51	NSCLC	cT4N0M0	Carboplatin + Paclitaxel + 50 Gy	5	VATS first	Lateral + Supine	1^st^ rib + Subcl. vein	ypT3N0M0	17	None	16	yes
	M	52	NSCLC	cT4N1M0	Carboplatin + Paclitaxel + 40 Gy	3	VATS first	Lateral + Supine	Clavicle + 1^st^ + 2^nd^ ribs	ypT4N0M0	15	None	62	yes
Present case	F	50	AdenoCa	cT3N0M0	Cisplatin + Pemetrexed + 60 Gy	10	VATS first	Supine	1^st^ + 2^nd^ ribs	ypT0N0M0	9	Hyperpyrexia	6	yes

*G* gender, *F* female, *M* male, *Pleom. Carc* Pleomorphic carcinoma

## Case presentation

**Fig. 1:**
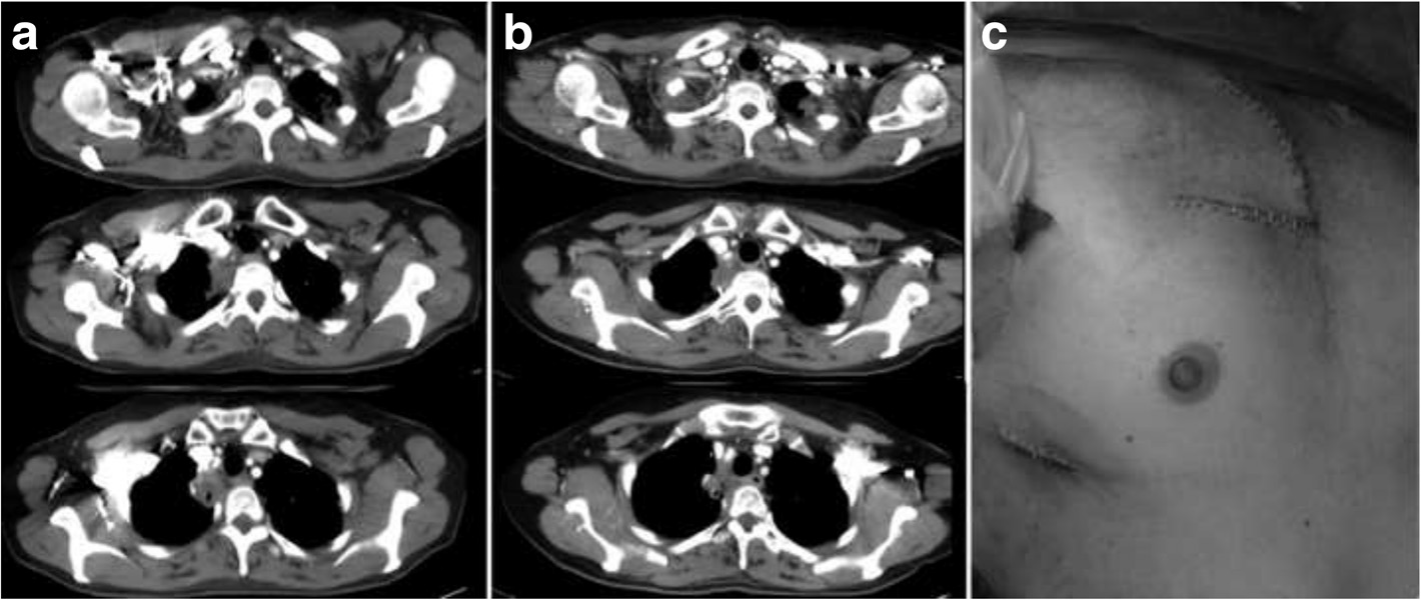
**a** Pre-induction therapy CT scan; **b** Post-induction therapy CT scan; **c** Modified Grunenwald incision and VATS incision

A 50 year-old woman with a 20 pack/years smoking history was referred to our Institution for right Pancoast tumor diagnosed as adenocarcinoma by fine needle aspiration cytology. The patient, presented with right arm pain, was staged with chest Computed Tomography (CT), brain CT and positron emission tomography (PET). The mediastinal and hilar nodal sampling at right thoracoscopy confirmed the N0 status diagnosed by CT and PET; in addition, the involvements of the right brachiocephalic and subclavian veins were highly suspected at the pleural inspection (stage cT4N0M0). The patient was treated with four cycles of cisplatin and Pemetrexed plus 60 Gy irradiation. Re-staging showed partial tumor regression at CT while PET scan became negative (Fig. 1a). The patient was placed in supine position and the surgery commenced with a thoracoscopy to evaluate the resectability through a 10-mm trocar in the eighth intercostal space on the midaxillary line. Once the VATS procedure was judged feasible, a 4 cm utility incision was made in the fourth intercostal space anteriorly to the latissimus dorsi muscle; an additional 10-mm incision was created posteriorly to the first incision. After adhesiolysis and dissection of the caudal mediastinal lymph-node stations, the upper lobe vessels were individually divided by staplers. Afterward, a limited C-shaped contra-incision was performed along the anterior border of the right sternocleidomastoid muscle and extended parallel to the second intercostal space. To preserve the sternoclavicular joint a vertical transmanubrial incision was made to reach the second intercostal space with a 5–6 cm extension to the right side (Fig. 1). Despite firm adhesions, there were no vascular or nervous infiltration and the apical dissection was considered complete after the resection of the anterior part of the first and second ribs, which were indissociable from the lung tumor. Through VATS accesses, sections of the right superior bronchus and the remaining fissure completed the lobectomy; the “en bloc” specimen was extracted through the apical incision. After the completion of lymphadenectomy, the sternum was sutured with metallic stitches and the anterior defect of the thoracic wall was repaired with a synthetic patch (5 cm in diameter).

Postoperative pain never excided 4 on the visual analog scale; neither intraoperative nor postoperative complications were observed and the patient was discharged 9 days after surgery (pathological result: ypT0N0M0). At the twelve-month follow-up the patient was free from disease and post-thoracotomy syndrome.

## Discussion and conclusions

In our opinion, the VATS procedure had several theoretical advantages. Rib retractors, known source of pain, were not used in any of the incisions; lobectomy was conducted through well-known accesses avoiding the uncommon transmanubrial adit for lobar dissection; Grunenwald contra-incision was shortened; unnecessary resection of thoracic wall was avoided. The “VATS observation first” has the advantage to exclude previously undetected pleural dissemination and to precisely define the tumor location; for instance, a needle inserted through the chest wall under endoscopic vision can help in focusing and minimizing the resection of rib segments. The majority of Authors change the patients’ position during the operation; on the contrary, we experienced a simple lobar dissection with the patient in the supine position: adopting the anterior approach to the hilar structures (Copenhagen approach), there is no reason to move the parenchyma from the position that it takes naturally after exclusion from the ventilation. In addition, we avoided the time-consuming maneuvers for patient repositioning (not less than 30 min in our theater) and the risk of endotracheal tube displacement.

We modified the Grunenwald incision extending the sternal section to the second intercostal space and reducing the lateral extension on the caudal edge; such variation was created in order to guarantee an easy access to great venous vessels.

Despite the experiences in hybrid approach to anterior Pancoast tumors are limited and technical details are inhomogeneous, it is possible to argue that the mini-invasive surgery could be effective on these patients. We strongly support the “VATS observation first” philosophy and patient supine position to face anterior Pancoast tumor with hybrid techniques. Further studies are advisable in order to define the real advantage of hybrid approaches on open surgery.

## Consent

A written consent for publication was obtained from the patient.
